# Skin Temperatures of Back or Neck Are Better Than Abdomen for Indication of Average Proximal Skin Temperature During Sleep of School-Aged Children

**DOI:** 10.3389/fpsyt.2020.494528

**Published:** 2020-09-18

**Authors:** Véronique Bach, Chris R. Abbiss, Jean-Pierre Libert, Susan M. McCabe

**Affiliations:** ^1^ Peritox, UMR_I 01, University of Picardy Jules Verne, Amiens, France; ^2^ School of Medical and Health Sciences, Edith Cowan University, Joondalup, WA, Australia

**Keywords:** children, sleep, thermoregulation, skin temperature, home setting

## Abstract

**Purpose:**

The tight association between sleep, body temperature regulation, and patterns of skin temperature change highlights the necessity for accurate and valid assessment of skin temperatures during sleep. With increased interest in this functional relationship in infants and children, it is important to identify where to best measure proximal skin temperature and whether it is possible to reduce the number of sites of measures, in order to limit the experimental effects in natural settings. Thus, the aim of this study was to determine the most suitable single skin temperature sites for representation of average proximal skin temperature during sleep of school aged children.

**Methods:**

Statistical analyses were applied to skin temperature data of 22 children, aged 6 to 12 years, measured over four consecutive school nights in their home settings, to compare single site measures of abdomen, back, neck, forehead and subclavicular skin temperatures (local temperatures) with average proximal skin temperatures.

**Results:**

Abdomen and forehead skin temperatures were significantly different (respectively higher and lower) to the other local proximal temperatures and to average proximal skin temperatures. Moreover, the time pattern of forehead temperature was very different from that of the other local temperatures.

**Conclusions:**

Local forehead and abdomen skin temperatures are least suitable as single site representations of average proximal skin temperatures in school aged children when considering both the level and the time course pattern of the temperature across the night. Conversely, back and neck temperatures provide most fitting representation of average proximal skin temperatures.

## Introduction

A close interaction between sleep and body temperature (T) has been shown to exist in animals and human neonates, children and adults. Two important consequences of this functional interaction are that sleep is regulated in phase with circadian body T regulation and that sleep disturbances are observed in association with thermoregulatory changes. Both sleep and thermoregulation are thought to be regulated by the preoptic area of the brain and anterior hypothalamus which further supports the functional interaction between these physiological processes [for a review, see (1)]. The association between sleep and T regulation highlights the necessity for accurate and valid assessment of body Ts within sleep research. Furthermore, the important effects of individuals’ physiological characteristics (age, sex, health conditions, pathologies and medications), activity (daily occupation, type and timing of exercise, diet, bathing, use of screens, change in body position) and environment (seasons, ambient lighting, household T and humidity, bed microclimate) on their sleep and body T regulation, mean that body T assessment must be feasible and reliable in natural settings.

Human body T assessment is often divided into the “core” and the “shell”. The core includes internal body organs such as muscles, lungs, heart, abdominal organs and brain and is typically measured *via* the rectum, esophagus or on the tympana. At rest, core body T remains relatively consistent between 36.5°C and 37.5°C. Conversely, the T of the peripheral shell (skin) is highly variable and heavily influenced by environmental conditions and mechanisms of heat gain and heat loss. Typically, metabolic heat produced within the core is transferred to the shell by internal conduction and by convection through blood flow. Blood flow is regulated differentially in each body region so that the skin T can differ drastically across regions. Areas with large arteries close to the skin surface (i.e. cheeks and the inguinal region) can be relatively hot (≥36.5°C), while limb skin T (i.e. ~33.5°C to 36.5°C at rest) and body extremities (<33.5°C at rest) can be much cooler.

In order to maintain body homeothermia, metabolic heat is lost to the environment through conduction, convection, radiation and evaporation and is modulated predominately by local skin blood flow and sweat rate. Heat exchange with the environment is dependent on the skin to environment T gradient. Cutaneous (skin) vasodilation and vasoconstriction play important roles in regulating skin blood flow to balance body heat loss and conservation. Distal body segments, and particularly the hands and feet, are characterized by many arterio-venous anastomoses [AVAs, shunts between arterioles and venules, which are innervated by the sympathetic constrictor neurons (2)] which adjust blood flow through the skin. Lyon et al. (3) have suggested that during development, the feet constitute the first body site to display vasoconstriction in term neonates. Early research (4) has shown that hand circulatory flow increases 44% when increased from 31°C to 35°C through water immersion. Changes in peripheral vasomotricity through AVAs can be assessed by the difference of skin Ts between the distal regions and proximal regions (assumed to devoid of AVAs), and are expressed as the distal-to-proximal skin T gradient (DPG**;** distal T minus proximal T). Therefore, even though core T provides fundamental thermal information to the hypothalamic regulating system, skin T plays a critical role in thermoregulation when ambient Ts varies [thermal transient (5)]. This is particularly true when considering the interaction between thermoregulatory processes and sleep-wake cycle regulation.

Sleep is regulated in phase with the circadian body T rhythm. From studies in adults, it has been shown that sleep onset usually occurs at or near the maximum rate of decline of core (rectal) T (6, 7). Conversely, morning awakening is associated with increases in core T. Magnussen [cited in (8)] suggested an autonomic (vegetative) sleep preparedness [starting around 100 min before sleep onset (8)] beginning with skin vasodilation. Behaviors before sleep and at bedtime facilitate vasodilation, mainly at the distal parts of the body. Distal skin vasodilation dissipates body heat and therefore leads to core T decrease, even when the person is clothed and covered. Thus, the patterns of distal (feet and hands, or wrist) skin T change are opposite to those of core T during the sleep-wake cycle (9–11). The patterns of proximal skin T change are intermediate, between the distal and the core T time patterns. Accordingly, during wake, the distal-to-proximal T gradient (DPG) is typically a negative value, with distal T lower than proximal T. The DPG increases (less negative, distal T rising toward proximal T) before sleep onset and during the first part of the night due to a more rapid increase in distal T than in proximal T. This leads to a “completely relaxed, one-compartment body” state [i.e. when DPG = 0°C, i.e., disappearance of the thermoregulatory shell (12)]. This is consistent with the observation that in preterm neonates homogenization between proximal and distal skin Ts was related to more rapid sleep onset (13). DPG can therefore be considered as a marker for skin thermal homogenization between the distal and the proximal regions of the body. Conversely, DPG decreases (more negative) towards wake and in the first part of the day (14). In this causal relationship, sleep is initiated as a consequence of the distal skin heat loss *per se* rather than of core T decrease (15), and skin T and the DPG might act as an input signal for the regulation of the sleep-wake cycle. Indeed, Kräuchi et al. (16) have demonstrated that the DPG is a better predictor of sleep onset than other measures, such as changes in core body T, melatonin level, heart rate, or subjective sleepiness rating. Subsequent studies have shown that the greater the DPG increase, the shorter the sleep onset latency (17, 18). Interestingly, alterations of body T pattern have been observed when sleep is compromised, in people with insomnia (19), narcolepsy (18) or children with bipolar disorder (20). Related to this, Boulant and Hardy (21) have shown that skin Ts modulate the firing rate of warm-sensitive neurons in the preoptic area and anterior hypothalamus, which are postulated as sleep-promoting signals. The neuronal activity of these warm-sensitive neurons increases at sleep onset and decreases prior to awakening and during wakefulness (22).

Studies performed on school-aged children sleeping in their natural settings showed that DPG [T_calf_ – T_subclavicular_ (20), T_feet_ – T_subclavicular, abdomen_ (23)] increased before and after sleep onset. Similar time patterns are also observed in preschool children (mean age: 4 years) (24), in 4 to 9 months old infants (25) and even in preterm neonates (13, 26). Although the usability of DPG to predict rate of sleep onset is reported as limited in infants (25) results show that, as in studies of adults, the larger the distal vasodilation, the more rapid the sleep onset (13). Also consistent with those shown in adults, the results of these studies of infants and children show that proximal and distal skin Ts do not share the same time patterns. Distal Ts have larger variation than proximal Ts, particularly in the initial hours from bedtime, leading up to and after sleep onset.

These results highlight the key role of skin Ts in the sleep wake-cycle, and point to the value of measuring skin Ts and DPG to complement polysomnography or actigraphy in sleep studies. Indeed, consideration of skin Ts goes beyond enhanced sleep assessment. It has been shown that it is possible to promote sleep initiation and maintenance with slight manipulations of distal and proximal skin Ts (producing changes that remain within the everyday circadian range) before and during sleep (27, 28). Experimentally induced peripheral vasodilation to enhance heat loss can be obtained with thermal manipulations (such as warm foot bath, use of hot water bottle) as well as non-thermal manipulations (e.g., lying down, closing eyes, active relaxation techniques) (for a review, see (29, 30). To the best of our knowledge, the use of deliberate slight thermal manipulation in order to improve sleep has never been studied in infants and children, despite its great interest. Notably, the relative importance of skin T (vs. core body T) in thermoregulation is understood to be even greater in infants and children than it is in adults, because of a higher density of skin thermoreceptors per surface unit and greater surface area (skin) to body mass (core) ratio [345 cm²/kg in 9–11 years old boys vs. 263 cm²/kg in young adults (31)] which enhance and accelerate the body heat exchange.

Reflecting the broad recognition of the importance of proximal and distal skin Ts in sleep research, various body sites have been used for measures of distal and proximal skin Ts (**Table 1**). To date, the majority of studies that have measured local **distal T** (sometimes called “peripheral”) have measured T at the foot. Conversely, a considerable number of studies have also measured distal T at the hand, wrist or forearm. More rarely, distal T has been measured at the calf or on the leg. Average distal T (as opposed to local single distal T) is arithmetically averaged from various combinations of measures of T at distal regions.

**Table 1:** Reported single and combined body sites for measures of distal skin T, proximal skin T and DPG.

Distal temperature		
Local distal temperature		
Foot	One foot, or average value of the right and left feet	(13, 23, 25, 32–36)
Hand	Measured variously at back of the hand(s), one or both middle fingers	(37, 38)
	On the fingertip	(39, 40)
	Thumb	(15)
Wrist		(11)
Forearm		(41)
Calf		(20)
Leg		(41)
**Average distal T**		
Combinations of measures of T at the hands (fingertip, finger, palm) or feet (instep, big toe)		(42)
Hands and feet		(18, 43–45)
Wrist and feet		(46)
Wrists and ankles		(47)
Extensive combination of ankles, calves, thighs, fingers, wrists and forearms		(48)
**Proximal temperature**		
**Local proximal temperature**		
Trunk	Subclavicular	(24, 45, 49)
	Sternum	(45, 46)
	Flank	(41)
	Axilla	(50)
	Abdomen	(25, 26, 37)
	Back	(51)
**Average proximal temperature**		
Pectoral and abdomen		(13)
Left and right subclavicular regions and sternum		(46)
Left and right subclavicular regions and sternum and thigh(s), abdomen, subclavicular region(s) and midthigh(s)		(18, 43–45, 52)
Subclavicular, sternal, back shoulders and spinal cross regions	Could be distinguished into back (shoulders and spinal cross) and front (subclavicular and sternum) regions.	(48)
**DPG**		
**Using single Ts**		
Proximal T not located on the trunk	Forehead and flank as proximal Ts and arm or leg as distal Ts,	(41)
	T_middle finger fingertip_ – T_forearm_	(39)
	T_thumb_ – T_forearm_	(15)
	Upper part of the body: T_ear lobe_ – T_mastoid_ middle part of the body: T_hand_ – T_arm_ lower part of the body: T_foot_ – T_leg_	(42)
Proximal T located on the trunk	T_foot_ – T_torso_	(53)
	T_foot_ – T_chest_	(24)
	T_calf_ – T_subclavicular_	(20)
	T_foot_ – T_abdomen_	(3, 25)
**Between average proximal and average distal temperatures**	T_wrists and ankle_ – T_clavicular and sternal_	(47)
	T_wrists and feet_ – T_subclavicular and sternal_	(46)
	T_hands and feet_ – T_subclavicular, thigh, stomach and forehead_	(14, 16, 54)
	T_hands, feet_ – T_subclavicular, thigh, abdomen_	(44)
	T_ankles, calves, thighs, fingers, wrists and forearms_ – T_abdomen, subclavicular region and midthigh_	(48, 52)
	T_ankles, calves, thighs, fingers, wrists and forearms_ – T_subclavicular, sternal, back shoulders and spinal cross regions_	(48)

**Proximal skin T** is more difficult to define. Proximal regions are most simply identified as those that are not distal, and proximal T usually refers to skin sites on the trunk (15). It has been identified that there are no or few AVAs in the skin of the chest (55), and the same is assumed for the rest of the trunk, with those regions accordingly considered not to play an important role in thermoregulatory heat exchanges (56). Some authors have included head regions [forehead (9, 41)] because no or few AVAs are observed on the forehead (55) but this is controversial (57). Moreover, regions supposed to have fewer AVAs are sometimes included in the proximal regions such as thigh (9) or forearm (15, 39), even though forearm is considered as distal region in other studies [**Table 1** (41)]. As for distal T, proximal T can be analyzed from a single site or as an average across several sites. Sites for single measurement typically include the trunk, including axilla, which is sometimes considered to be a reliable alternative of rectal T measure (58)), and abdomen, which is often chosen because of its special characteristics (position near the well irrigated liver, with non AVAs). In neonates under continuous monitoring of body T for the regulation of the incubator air T, abdominal T is often considered to be a good and non-invasive indicator of core T (3). More rarely, back is considered as a single site for proximal T, but in these studies T is measured on the mattress in the back region (51).

Average proximal T is obtained from various arithmetic averages of local proximal regions. The average values are calculated without (9) or with (16) weighting factors, which are roughly calculated according to each skin surface area over which the sensor is placed relative to the body surface area of the segment as a whole. It should be noted that this may differ for children and infants since proportions and skin surface area of each body segment change with development.

Given the vast possibilities for determining proximal or distal T measurement, **DPG** **(distal T minus proximal T)** may be calculated in different ways. Using single Ts, calculations have included proximal T which is not located on the trunk (forehead, flank, limbs) even though they have been shown to show similar time pattern as hands or feet T (13). Unique DPGs have been calculated between upper (T_ear lobe_ – T_mastoid_), middle (T_hand_ – T_arm_) and lower body (T_foot_ – T_leg_) regions, and exhibit anti-correlated fluctuations in the hand-to-arm and foot-to-leg gradients (42). Most clear-cut formulas include trunk proximal T with T_foot_ – T_abdomen_ more often used. The DPG can also be obtained from the difference between average distal and average proximal skin Ts, with a range of calculations reported.

It is clear that there are a range of possible sites and formulas for the measurement of proximal T and, to a lesser extent, distal T. Logically, it could be assumed that proximal T may be most accurately assessed as the average of multiple measures, from all over the proximal regions. That is, the more numerous the measurements, the more accurate the calculation of average proximal T. Indeed, some studies have calculated skin T from averages over 65 locations for a “full-body thermography” (59) or with skin infra-red thermography (60). Conversely, the use of multiple sites is at odds with practical considerations. This is of particular interest when considering measures in home settings, where the subject, relatives or caregivers are required to be accurate in their timing and placement of the sensors. As well as reducing participants’ preparation time and reducing possible impact on their sleep, reducing the number of sensors reduces the risk of errors of sensor location.

It may be practical to reduce the number of sensors used in infants and young children. Moreover, for those with behavioral or cognitive impairments, it would be safer to locate sensors at body sites where they are least likely to be tampered with or removed. With increased interest in the functional relationship between thermoregulation and sleep in infants and children, it is important to determine the accuracy of measures when fewer skin T sites are used with these populations. Morphological and thermoregulatory differences prevent extrapolation from results of studies of adults. Thus, the purpose of the present study was to compare single locations for measure of proximal T with that determined by average of proximal T sites. Special interest was given to back T as the preferred site, for the abovementioned reasons, using sleep and skin T data of children aged 6 to 12 years.

## Methods

**Figure 1:**
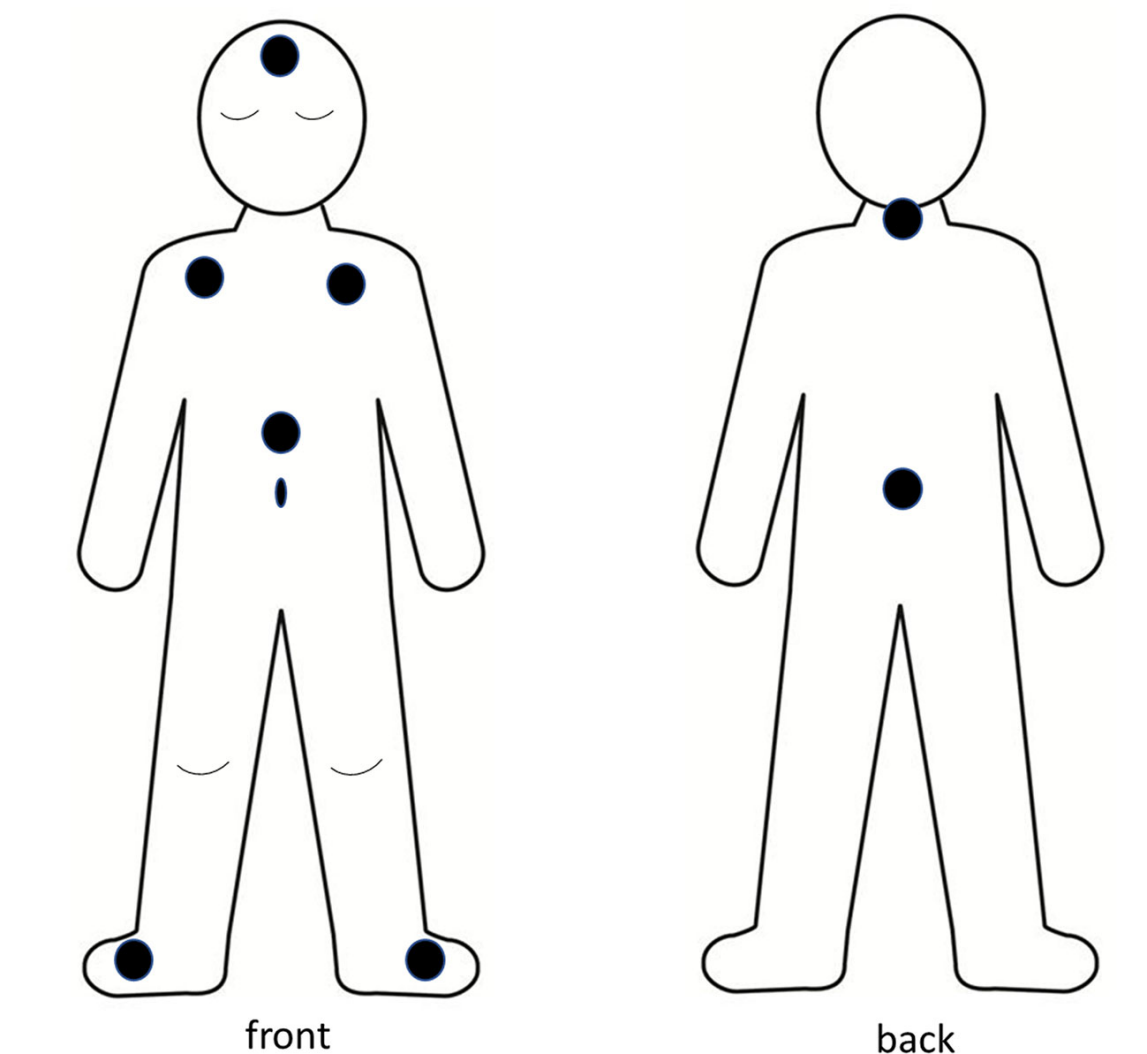
Body schema of location of iButton sensors.

The present study is additional analysis of data which were collected as part of a study of reliability and patterns of skin T and sleep in 22 healthy school aged children, aged 6 to 12 years (mean, SD: 9 years 6 months ± 1 year 10 months), over four consecutive school nights in their home settings (23). Prior to participation, children provided written assent, and their parents provided written consent based on written information and verbal explanation of the requirements and possible risks associated with the study. The study was approved by Edith Cowan University Human Research Ethics Committee. Data collection was conducted in Perth Western Australia during the months of May to October (autumn to spring). The overnight bedroom Ts ranged from 15.6°C to 21.5°C (mean 18.5°C ± 1.4°C), bedroom humidity ranged from 49.2% to 75.4% (mean 63.8 ± 6.89%), and the bedroom ambient light ranged from 7.1 lux to 18.2 lux (mean 10.3 ± 2.7 lux). In addition to measures of sleep habits, perceptions of thermal comfort, core body T and ambient bedroom light and T the children’s sleep was measured through use of actigraphy (Actigraph GT3X+ activity monitors, Actigraph, FL, USA) and parent log books [for full description, see (23)]. Their skin Ts were measured through use of Thermochron iButtons (DS1922L, Maxim/Dallas Semiconductor Corp., USA). The iButtons are specified to have accuracy of ±0.5°C in a range of 10°C to +65°C (www.maximintegrated.com). They have been found to provide valid measures of human skin Ts in natural settings (61). The iButtons were pre-set to record every 5 min, at high resolution (0.0625°C). Parents were instructed to place iButtons onto their child’s skin 1 h before bedtime and to remove them on morning waking. Reflecting methods reported in other studies of human skin T (47, 62–64) the iButtons were attached to the children’s skin using air-permeable adhesive skin tape (Fixomull, Beiersdorf, Hamburg, Germany). Eight skin sites were used, with iButtons attached at left and right feet (T_feet_), abdomen (T_abdo_), left and right subclaviculae (T_clav_ = averaged from T_right clav_ and T_left clav_), forehead (T_forehead_), back of neck (T_neck_), and central back area (T_back_) (**Figure 1**). Notably, the study did not include measures of hand or finger. The decision to omit hand or finger T was based on safety considerations, to avoid risk of inadvertent ingestion of the small, battery sized sensors. The skin T data were formatted using Excel (Microsoft, 2016), in alignment with reported bedtimes for each child, each night. All skin Ts had high night to night reliability, as previously demonstrated (23).

### Data Analyses

For the current study, several formulas were used to calculate average proximal T, with different weighting coefficients. These reflect formulas most commonly used in published studies, and are shown as i) Formula 1, with a weighted formula (Tw) modified from Kräuchi et al. (14) using abdomen, right and left subclaviculae (averaged) and forehead Ts; ii) T4(R=L) Formula 2, using averaged values of left and right subclavicular Ts, abdominal T and back T; iii) T4 Formula 3, with the same local Ts, but attributing the same weighting factor for each of the 4 measures; and iv) T3 Formula 4, using a more typical formula for proximal Ts (right and left subclaviculae, abdomen) (23)

$$\begin{array}{l} Formula\;1:\\ Tw=0.45\times T_{abdo}+0.407\times T_{clav}+0.143\times T_{forehead} \end{array}$$

$$\begin{array}{l} Formula\;2:\;\\ T4(R=L)=0.33\times T_{abdo}+0.33\times T_{clav}+0.33\times T_{back} \end{array}$$

$$\begin{array}{l} Formula\;3:\;\\ T4=0.25\times T_{abdo}+0.25\times T_{right\;clav}+0.25\times T_{left\;clav}+0.25\times T_{back} \end{array}$$

$$\begin{array}{l} Formula\;4:\;\\ T3=0.33\times T_{abdo}+0.33\times T_{right\;clav}+0.33\times T_{left\;clav} \end{array}$$

In addition, distal to proximal gradients (DPGs) were calculated using each local proximal T: T_feet_ – T_local proximal_.

### Statistical Analyses

Normal data distribution was checked using a Kolmogorov-Smirnov test. Local proximal Ts (T_abdo_, T_clav_, T_back_, T_neck_, T_forehead_), average proximal Ts (Tw, T4(R=L), T4 and T3) and variations of DPG (distal T - local proximal T) were compared using a two way repeated measures analysis of variance (Statview 5.0) to analyze the site and formula effects. Where significance was observed posthoc analysis was performed using Posthoc test of Least Significant Difference (PLSD).

Each of the local proximal Ts (T_abdo_, T_clav_, T_back_, T_neck_, T_forehead_) was compared to each of the average proximal Ts (Tw, T4(R=L), T4 and T3) by paired t-tests. This analysis was split or not into time of night and/or night.

Results are given as average values ± SEM. Significance was considered at p < 0.05.

## Results

### Local Proximal Skin Temperatures

**Figure 2:**
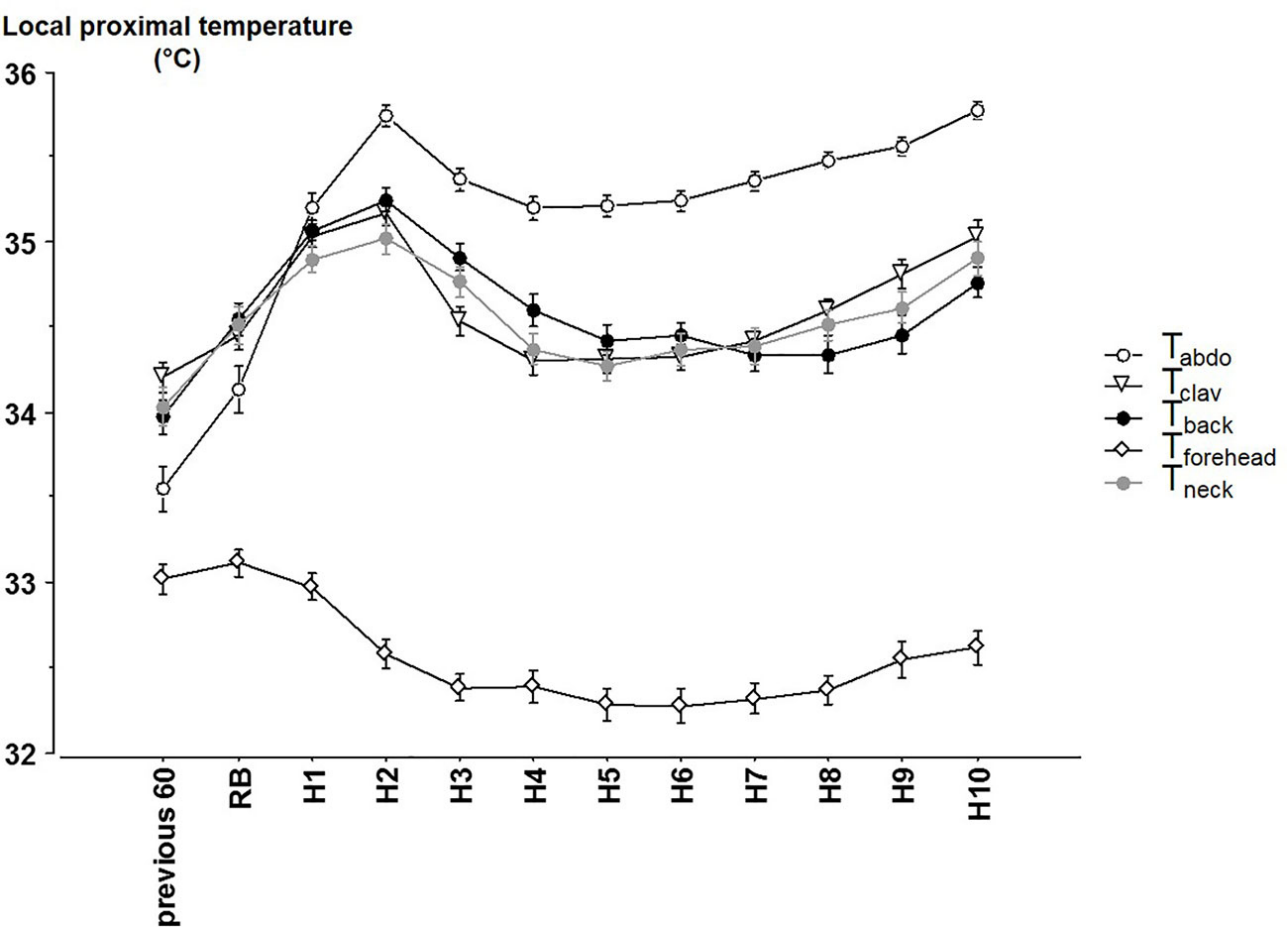
The different local proximal skin temperatures (mean ± SEM) from 60 min before reported bedtime, at reported bedtime (RB) and at hourly timepoints after reported bedtime. abdo: abdominal Temperature, clav: average of the right and left clavicular temperatures. Previous 60 = during the 60 min before reported bedtime (RB), H1 to H10 hours in bed.

Significant time, site and interaction site x time effects were observed for local proximal skin T (**Figure 2**) (all p < 0.001). T_abdo_ was greater and T_forehead_ lower than other Ts (always p < 0.001). T_clav_, T_back_ and T_neck_ did not significantly differ from each other throughout the night. The significant site x time interaction (p < 0.001) reveals that the time course pattern may differ from one T to another, and that the differences between Ts are not kept constant throughout the night. Further analyses show two different patterns across the night (**Table 2**). T_abdo_, T_clav_, T_back_, and T_neck_ significantly increased across each time point from reported bedtime until H2 and then decreased until H4. A final increase was then observed which began earlier for T_abdo_ (H6), later for T_clav_ (H7) and even later for T_back_ and T_neck_ (H9). Conversely, T_forehead_ significantly decreased from reported bedtime to H3 and finally increased from H8 to the end of the night. As a result, T_abdo_ was initially significantly lower (0.53°C in average) than all the other Ts (at previous 60 min, and at RB) and thereafter was significantly higher than these Ts (from H1 to H10, +0.76°C, 0.75°C, 0.80°C when compared to T_clav_, T_back_, T_neck_, 2.94°C when compared to T_forehead_). In contrast, T_forehead_ was on average 2°C lower than the other Ts. T_back_ was sometimes significantly higher (H3 and H4) or lower (previous 60 min, and during the last part of the night from H8 to H10) than T_clav_. The greatest difference was observed at H3 (+0.37°C). During the last 3 h of the night, the difference between T_back_ and T_clav_ was 0.30°C, on average (p < 0.04). The other comparisons did not show any significant difference between T_neck_ on one hand and T_clav_ or T_back_ on the other.

**Table 2:** Comparisons of each local proximal skin temperature (T_abdo_, T_clav_, T_back_, T_neck_, T_forehead_) from 1 h to the next one.

	previous 60 min vs RB	RB vs H1	H1 vs H2	H2 vs H3	H3 vs H4	H4 vs H5	H5 vs H6	H6 vs H7	H7 vs H8	H8 vs H9	H9 vs H10
**T_abdo_**											
**T_clav_**											
**T_back_**											
**T_neck_**											
**T_forehead_**											

Previous 60 = during the 60 min before reported bedtime (RB), H1 to H10 hours in bed. Light grey indicates significant increase (p < 0.05), dark grey indicates significant decrease, white indicates NS comparisons.

### Average Proximal Skin Temperatures

**Figure 3:**
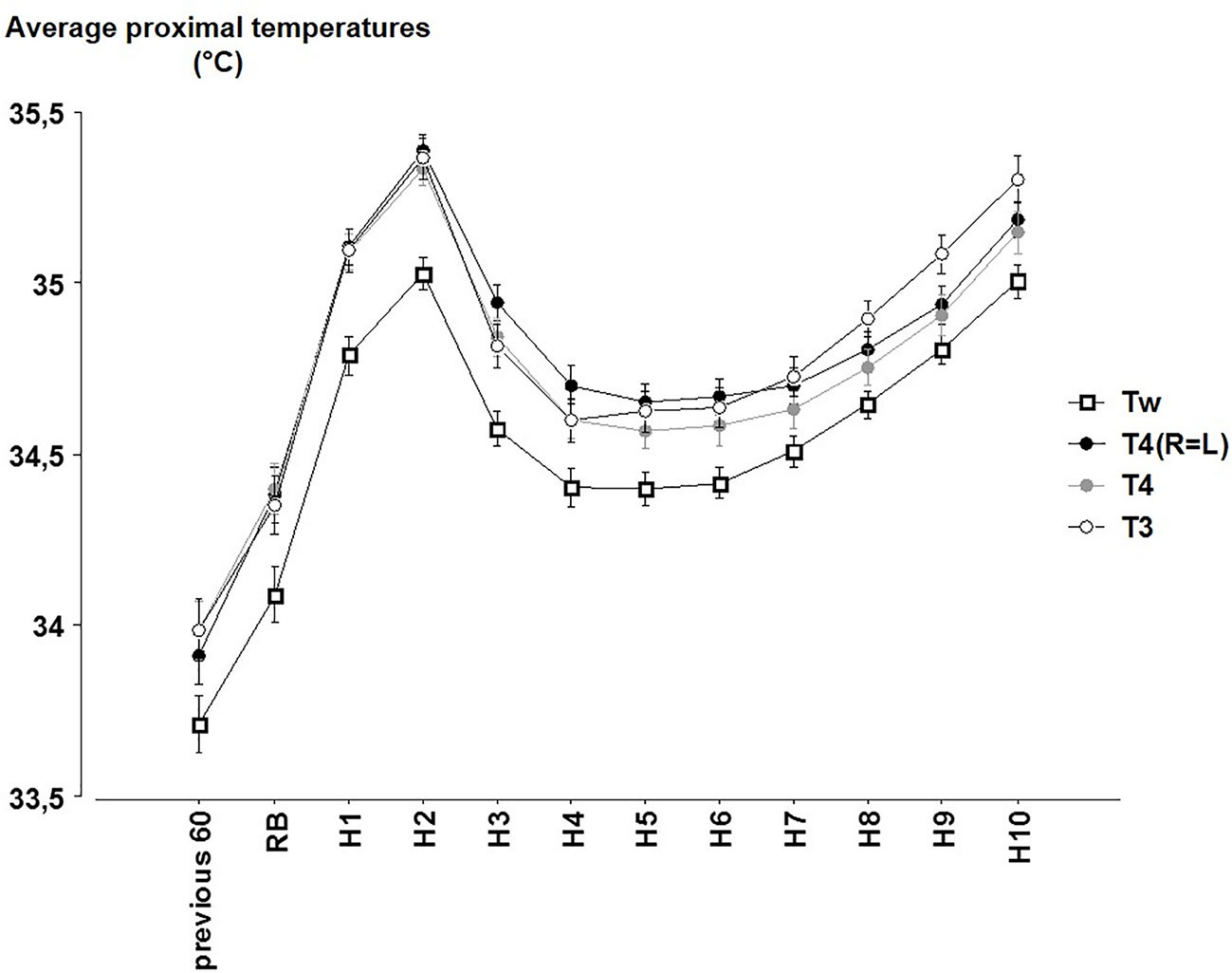
The hourly averages of the different proximal temperatures (Tw = 0.45 T_abdo_ + 0.407 T_clav_ + 0.143 T_forehead_; T4(R=L) = 0.33 T_abdo_ + 0.33 T_clav_ + 0.33 T_back_; T4 = 0.25 T_abdo_ + 0.25 T_right clav_ + 0.25 T_left clav_ + 0.25 T_back_; T3 = 0.33 T_abdo_ + 0.33 T_right clav_ + 0.33 T_left clav_) (mean ± SEM) according to the time (Previous 60 = during the 60 min before reported bedtime (RB). H1 to H10 hours in bed).

Significant effects were observed across time (p < 0.001), between each of the formula for proximal T (p < 0.001; **Figure 3**) and an interaction for time x formula (p < 0.001). Tw, which takes into account the lowest skin T of the forehead, was always significantly lower than the other averages of proximal T (in average −0.24°C, always, p < 0.001).

Comparisons between T4(R=L), T4 and T3 are sometimes significant or not according to the time. T4 and T4(R=L) values significantly differ across most time points (except at RB and H1) whereas exclusion or inclusion of the back T in the average (T4 or T4(R=L) vs. T3) led to significant differences during the last part of the night (from H5 to H10). These differences however remain lower than 0.16°C (between T4 and T3 at H9, p < 0.001).

### Distal to Proximal Gradient

**Figure 4:**
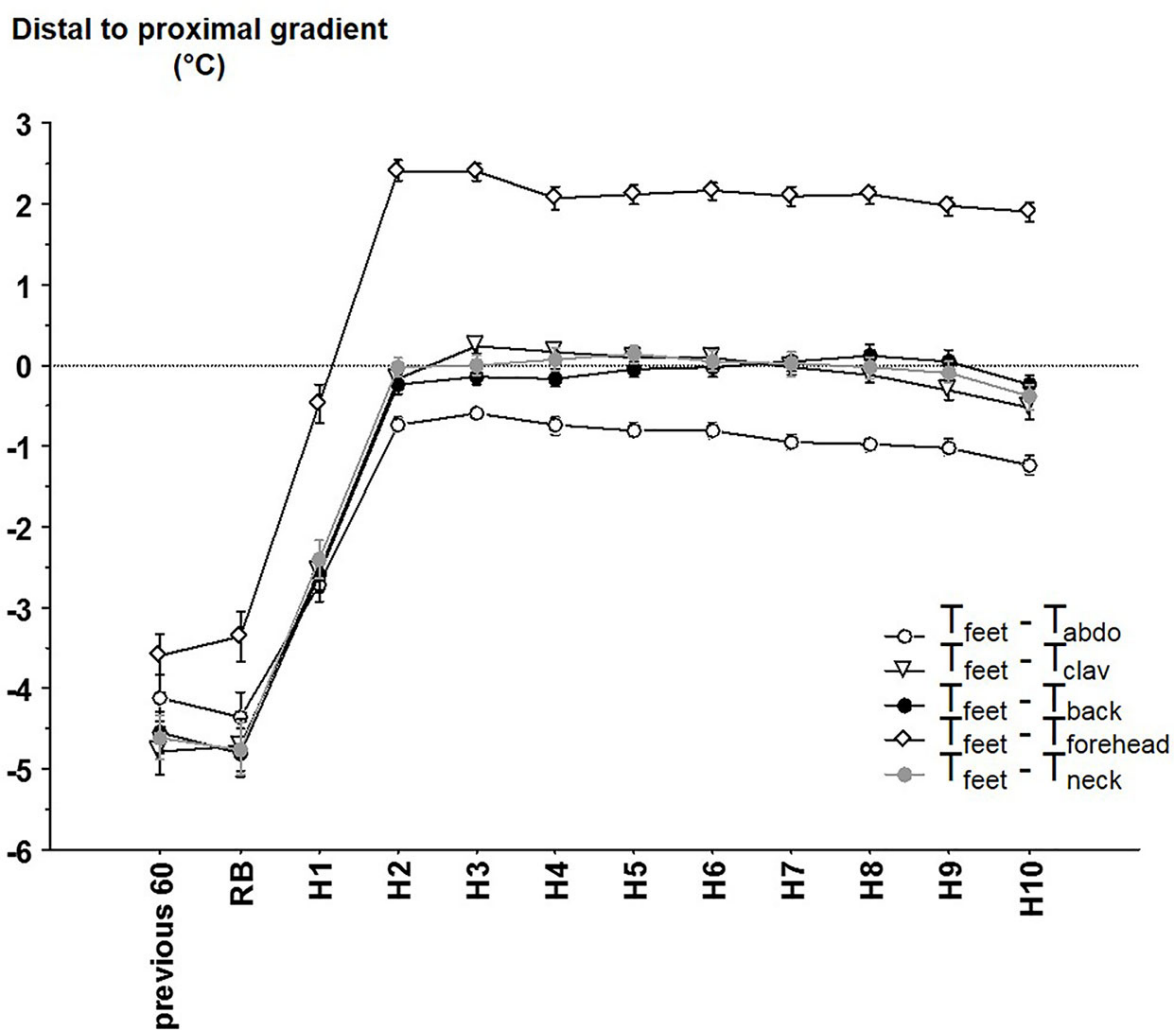
The different distal-to-proximal skin temperature gradient (mean ± SEM) calculated between distal (i.e. feet) temperature and abdominal temperature (T_abdo_), average right and left clavicular temperature (T_clav_), back temperature (T_back_), forehead temperature (T_forehead_), neck temperature (T_neck_) according to the time. Previous 60 = during the 60 min before reported bedtime (RB), H1 to H10 hours in bed.

Distal to proximal gradients were calculated using the different local proximal T (T_feet_ – local proximal T; **Figure 4**). Consistent with the previous results on local proximal Ts, distal to proximal gradients exhibit significant time (p < 0.001), local proximal T (p < 0.001) and time x local proximal T (p < 0.001) effects. DPG calculated with the T_forehead_ was also significantly different from DPG calculated from other local proximal sites (2.15°C in average). DPG calculated with T_abdo_ was also significantly different (except for the comparison with T_back_ at H1): the average difference with DPG calculated with T_clav_, T_back_ and T_neck_ was 0.56°C). The other comparisons between the DPG calculated with T_clav_, T_back_ and T_neck_ were mainly non-significant.

### Local Proximal T as Indicator for Average Proximal Temperature

**T_abdo_** was always significantly different from average proximal T, regardless of the average proximal T it was compared to (except comparison with Tw at RB). This was observed when split according to time (measured on hour to hour basis), i.e. 98% of the comparisons (**Table 3**), and in 84% (162/192 significant comparisons) when split according to time and night. During the 60 min before bedtime, and at RB, T_abdo_ was lower than the average values, whereas during the night, it was higher.

**Table 3:** Results of the paired comparisons between each of the local proximal Ts (T_abdo_, T_clav_, T_back_, T_neck_, T_forehead_) with each of the average proximal Ts (Tw, T4(R=L), T4, T3).

		previous 60 min	RB	H1	H2	H3	H4	H5	H6	H7	H8	H9	H10
**T_abdo_*vs***	**Tw**												
	**T4(R=L)**												
	**T4**												
	**T3**												
**T_clav_*vs***	**Tw**												
	**T4(R=L)**												
	**T4**												
	**T3**												
**T_back_*vs***	**Tw**												
	**T4(R=L)**												
	**T4**												
	**T3**												
**T_neck_*vs***	**Tw**												
	**T4(R=L)**												
	**T4**												
	**T3**												
**T_forehead_*vs***	**Tw**												
	**T4(R=L)**												
	**T4**												
	**T3**												

Light grey indicates that local proximal T was higher than the average proximal T (p < 0.05), dark grey indicates that local proximal T was lower than the average proximal T, white indicates NS comparisons.

Similar conclusion can be drawn for **T_forehead_** which was always significantly lower than the average values of proximal T (100% when split into time, and 100%=192/192 of the split into time and night).

**T_clav_** exhibited intermediate results (77% and 55%=105/192) being at the beginning of the measurements higher than the average values and then lower.

**T_back_** and **T_neck_** showed the best results, with respectively only 58% and 31% (60/192) for T_back_ and 61% and 19% (36/192) for T_neck_ of the comparisons being significantly different from the average proximal T. T_back_ showed a specific pattern with near-average proximal T values during the first part of the night (from H1 to H5) and deviation from these values during the second part of the night, indicating a specific pattern of T_back_ during this part of the night when compared with the other local proximal Ts.

## Discussion

The main purpose of the present study was to compare skin temperatures, measured at single proximal sites, with commonly used averages across multiple sites, to determine the most apt single site for representation of proximal T. A secondary aim was to examine the effect on calculations of average proximal T of including or excluding the specific local Ts. The main finding from this study was that that T_back_, T_neck_ and to a lesser extent T_clav_ are more suitable indicators of average proximal T than T_abdo_, or T_forehead_. Additionally, we found that calculations which included T_forehead_ for average proximal T were significantly lower than the other formulas used for this measure. To our knowledge, this is the first study to identify T_back_ or T_neck_ as the most suitable single sites for representation of proximal T during sleep of school aged children, and to identify the significant effect of inclusion of T_forehead_ in calculations of average proximal T.

Average or local proximal Ts usually follow a pattern similar to that of core T in adults exposed to constant routine (9) as well as during their sleep wake cycle, which differs to that of distal T (14). Differences in distal T may result from passive blood flow modification in proximal regions lacking AVAs when distal regions vasoconstrict or vasodilate (9). In our study, only T_forehead_ is likely to have been consistent with core T pattern (not measured), while the pattern of other proximal Ts (local and averages) were similar to distal T (though of lower amplitude). Interestingly, Okamoto-Mizuno et al. (32) have observed that proximal T (chest) increased (as did distal T) in adults as well as in preschool infants around bedtime, but that age differences appeared after between infants (whose T_chest_ decreased though the night) and their mothers (whose T_chest_ remained almost constant until the end of the night). In contrast, in preterm neonates (26) as well as in 4 to 9 months old infants (25), T_abdo_ did not exhibit any notable change around sleep onset. Okamoto-Mizuno et al. (32) have suggested that these age differences may be explained by the fact that children, in contrast to adults, may rely more on proximal T than on distal T to lead core T decline during the sleep wake cycle. Such differences could result from vascular modifications: when exposed to hot environment, prepubertal boys were able to increase skin blood flow on the trunk (chest and back) (65) but also on the forehead (66) more than young men. In another study, these authors pointed out that cutaneous vascular conductance was larger on the forehead and the back (but not on the chest or the thigh) in boys than in young men (67). Finally, blood flow rate in the AVAs measured on the toe was also larger in 3 to 15 years old children compared to young or older adults (68). Interestingly, despite such differences regarding proximal T, DPG exhibits similar pattern between adults and infants, though of lower (more negative) value in children, indicating higher heterogeneity between the skin Ts when compared with adults (32).

There is currently no consensus in the literature on which local Ts should be taken into account to determine a proximal T, and to calculate DPG. Average proximal T is usually considered to be more reliable than its individual components as least when their rhythms are considered (9). However, for studies in natural settings, it would be of considerable practical advantage if a single site was determined as a sound indicator of average proximal T.

### Average Proximal T

It has been demonstrated in adults that a set of 3 sensors that were positioned either on abdomen, subclavicular areas and mid thighs but were not on homologous regions (i.e. a right/left counterpart) can provide a reliable estimate of proximal T (43). We deducted from this that proximal T measured with at least 3 sites on the trunk (i.e. left and right clavicular regions, used as single Ts or as an average one, abdomen, back) could be our gold-standards. We also calculated Tw using area weighting coefficients for clavicular, abdomen, back and forehead (modified from 14), although calculating the average proximal T by the means of such weighting factors is controversial since it does not take into account the differential distribution and sensitivity of the skin thermodetectors over the skin surface area (69).

Our results confirm the notion that the location of the skin T, as well as the choice of the weighting coefficients, will significantly change the reckoning of average proximal T. Importantly, the impact of the T_forehead_ in the average led to a −0.25°C difference with the other average proximal Ts (with a maximal gap of 0.37°C, which may be considered as moderately large). With exclusion of the T_forehead_ value from the average proximal T (consistently with the arguments developed below), it is notable that the differences between the remaining average values (T3, T4 and T4(R=L)) are only pronounced during the last part of the night, because of the specific time pattern of local proximal Ts, especially of T_back_. However, these differences remain at a low level (≤0.16°C), so that there is no important issue with respect to this choice.

### Local Proximal Ts

To provide sound indication of proximal T, a local T candidate should fulfill several criteria regarding the level and the time pattern across the sleep-wake cycle.

**Abdominal T** alone (25, 37) or included in an average formula (14, 16, 44, 48, 52, 54) has a clear preference in most studies of the literature for predicting proximal T and/or DPG. We observed that its time pattern is quite similar to other local proximal Ts, with a sharper increase between the 60 min period before recorded bedtime and H2 (+2.19°C). Part of this increase - as of the increases of the other local Ts that were measured under the bedding - is probably due to the attainment of a microclimate in the bed. Skin vasodilation around bedtime and sleep onset induces body heat losses that leads to core T decrease in naked as well as in covered subjects. For these latters, peripheral vasodilation allows the air T of the microclimate between the skin surface area and the covers to reach and remain constant at between 29 and 35°C (70, 71). The creation of an approximately thermoneutral microenvironment in the bed helps to protect the sleep stage structure (72). Interestingly bedding doesn’t seem to change the time pattern which is maintained for covered feet and uncovered hands throughout a 24-h routine protocol (9). Unfortunately, we did not measure the T inside the bed. The increase of T_abdo_ was however greater than that of the other trunk Ts (+0.97°C for T_clav_ and +1.28°C for T_back_) so that another phenomenon could intervene.

Comparisons with the different average proximal Ts pointed out that the level of T_abdo_ was always significantly lower (previous 60 min and RB, i.e. before sleep) or higher (from H1 to H10, i.e. probably during sleep; differences > 0.75°C) than the other local proximal Ts. In awake sitting adults, T_abdo_ was demonstrated to be higher than the other proximal Ts (subclavicular, thigh and forehead) (9). As a result, in our study DPG calculated with T_abdo_ was larger than those calculated with the other local proximal Ts (+0.56°C in average). This difference decreased during the last part of the night since the pattern for T_abdo_ was slightly different at the end of the night (beginning to increase at H6). Finally, T_abdo_ was always significantly different from all the average proximal Ts (whatever the formula), stressing the fact that T_abdo_ used alone cannot be considered as a good indicator of these averages assumed to be the gold standards, in contrast to the general belief. Longato et al. (43) have however reported that T_abdo_ should be integrated when calculating average proximal T, since the least accurate reconstruction for average proximal T occurred when the abdominal sensor was removed over the 5 sensors used.

### T_forehead_ and T_neck_

The head plays a major role in thermoregulation, not only because of the large skin relative surface area (73), lower thermal insulation (74) and higher thermosensitivity (75, 76) but also because of the presence of numerous AVAs, at least in the areas irrigated by the angular and facial arteries (55). This irrigation could provide a selective refreshment of the hypothalamus. We observed that the levels and the patterns for T_neck_ and T_forehead_ were very different. Differences in the heat exchanges (the forehead is consistently uncovered, with low tissue insulation, more often exposed to air over the bed; the neck is probably not covered by sheets and covers but may perhaps be covered by long hair and/or isolated by supine sleeping) may at least partially account for this discrepancy. Differences in the vasomotor control, irrigation (temporal artery for the forehead and carotid artery for the neck) and the fact that the forehead is devoid of arteriovenous anastomoses (55) and countercurrent mechanisms may also be involved and could explain that previous studies have demonstrated that forehead T would accurately reflect core T (77). However, this remains controversial (78).

Kräuchi and Wirtz-Justice (9) found, in a constant routine protocol (awake sitting subjects over a 24-h protocol), that T_forehead_ followed the same circadian pattern as T_rectal_, but at a lower level (33.58 ± 0.21 vs 36.77 ± 0.07°C, respectively). Even though we did not measure continuously core T, we can assume that our results could be consistent with also a lower level (32.58 ± 0.03°C vs T_tympanic_ = 36.44 ± 0.03°C).

The time pattern of T_forehead_, in contrast to the other local proximal Ts, did not continue to increase after reported bedtime. T_forehead_ was in correlation with ambient T of the bedroom (data not shown, see (23)), was on average 2°C below the other local Ts and was always significantly lower than all the average proximal Ts. These results suggest that T_forehead_ is not a suitable as a local measure of proximal T, nor as a part of calculations for average proximal T.

In contrast to T_forehead_, **T_neck_** showed much narrower difference from the other local proximal Ts and was less often significantly different from the average proximal Ts (see **Table 3**), so that T_neck_ could be a good indicator of proximal T. T_neck_ has also been measured as an indicator of head T when using a cooling pillow in hot environment (79).

### T_back_ and T_clav_

Both **T_back_** and **T_clav_** follow a waveform similar to that of distal T (data not shown, see (23)) but of lower magnitude. As such, our results do not reflect the finding that T_clav_ in adults under constant routine shares the time pattern as core T (9). In contrast to the other local proximal T, **T_back_** seems to fulfill all/most of the conditions to be a good indicator *per se* of average proximal T level, as well as patterns of change over time. Comparisons with average proximal Ts indicate that T_back_ and T_neck_ (see above) are good indicators of proximal Ts. This is also true, but to a lesser extent, for T_clav_, as featured by more significant differences when compared to average proximal Ts. It must be noted however, that at the end of the night, these local Ts adopt specific time patterns, leading to slight but significant differences between each other and with average proximal Ts.

The impact of prone or supine sleeping position on back or ventral T measurements could not be analyzed in our study. In supine sleeping adults, fluctuation of Ts across the time were reduced on the back body regions compared to the ventral Ts (59). However, another study concluded that “skin-mattress T” (i.e. T measured on the back when sleeping supine) was a reliable measure of core T at least in normothermic neonates (58). Indeed, in such situations, sleeping supine almost suppresses the heat flow between the skin and the mattress (the T of which is almost equal to back T), as a result, T_back_ is directly correlated to core T variations.

Moreover, as far as T_back_ (midline of the back, approximatively T11-T12) and T_neck_ (midline of neck, below hairline) are considered, age differences should be considered: in human neonates, brown adipose tissue (BAT, 2–6% of body mass) is located nearby the neck, in the interscapular region between the ribs and near the kidney. When exposed to cold environment, metabolic heat is produced in the BAT and its blood flow can increase up to one fourth of the cardiac input (80), maintaining a warm blood flow to the central nervous system and preventing brain cooling. As a result, the highest skin T is measured in the interscapular region, over the BAT [for a review, see (81)], and this T fell less than that of the other regional skin surface areas when the neonate is exposed to cool environment (82). Therefore, in this population, T measured nearby the BAT is rather an indicator of the increased activity of the BAT in response to cool challenge and/or of thermal-adaptive mechanisms when the challenge is prolonged (83).

### Challenges and Limitations

It is important to measure and understand the relationship between skin Ts and sleep in people of all ages. Because of the impact of behavioral and environmental factors on thermoregulation and sleep, it is important that measures take place in people’s natural settings. This creates challenges and limitations. The use of multiple sites for measures of skin T may enhance the reliability of data collection, by i) reducing risk of data loss due to inadvertent loss of iButtons and ii) accounting for the differences which may be observed across the night (due to body position change, displacement of bedcovers). However, use of multiple iButtons, with multiple skin sites, is likely to increase the risk of inaccurate placement of each iButton on the correct location. Furthermore, the time taken to apply iButtons to multiple sites may impact on the natural bedtime routines of individuals and families, and thus compromise the true nature of data collection in their home settings. It is important for researchers to understand the accuracy and reliability of single sites in comparison to multiple sites, so that the decision for each can be determined on true merit.

It is a limitation of this study that data are taken from just four school nights (Monday to Thursday). This was done to limit the variation between the children’s daily activities, and also with concern regarding the possible burden of participation on the children and families. It meant, however, that the effects of weekend variations in activity were not accounted for. It is possible that, with confidence in the accuracy and reliability of use of fewer iButtons, researchers could reasonably ask participants to undertake more nights of data collection, and across different seasons, for more valid measures in natural settings and with everyday routines. An additional limitation is the fact that daytime wakefulness period was not considered, except for the 1-h long period before recorded bedtime. It would be of great interest to analyze whether T_back_ and T_neck_ are good indicators of proximal T also during the daytime/activity period.

## Conclusions

There is an important relationship between sleep and body T, and both are affected by variation in individuals’ activities and environments. It is fundamental, then, that body Ts are measured during sleep studies in natural settings as well as in sleep laboratories. With improved knowledge of body T rhythms across the sleep wake cycle of infants and children, there is exciting opportunity towards future studies where slight manipulations of skin body Ts and/or non-thermal (behavioral, environmental) parameters may improve sleep initiation and maintenance. In light of the major role of sleep in health and development, this is of particular interest for infants and children, particularly those with particular sleep disturbances, health conditions and developmental or neurological disorders. It is important that researchers have confidence in data collection which places minimal burden on participants, without compromising accuracy and reliability. Thus, practical considerations would favor the use of fewer thermal sensors in home-based studies. Our results show that T_abdo_ is not a suitable indicator of average proximal T: the level is too high (overestimation of the T) and the pattern is different during the night (so the error of this estimation is not the same across the night). Similar conclusion can be drawn for T_forehead_, the pattern of which is more similar to that of core T. T_back_, T_neck_ and to a lesser extent T_clav_ are more reliable indicators of average proximal T when used as single sites.

## Data Availability Statement

The datasets for this article are not publicly available due to ethical requirements and the level of consent given by participants. Requests to access the datasets should be directed to [CA, c.abbiss@ecu.edu.au].

## Ethics Statement

The studies involving human participants were reviewed and approved by Edith Cowan University Human Research Ethics Committee. Written informed consent to participate in this study was provided by the participants’ legal guardian/next of kin.

## Author Contributions

SM and CA conceived the study. SM collected the data. VB and SM analyzed the data. All authors contributed to the article and approved the submitted version.

## Conflict of Interest

The authors declare that the research was conducted in the absence of any commercial or financial relationships that could be construed as a potential conflict of interest.

## Abbreviations

AVA, arterio-venous anastomoses; BAT, brown adipose tissue; DPG, distal to proximal gradient; SD, standard deviation; SEM, standard error of the mean; H1, H2, H3, H4, H5, H6, H7, H8, H9, H10, time (in hour) after reported bedtime; RB, reported bedtime; T, temperature; T3, T4, T4(R=L); Tw, average proximal temperatures; T_abdo_, abdominal temperature; T_clav_, clavicular temperature; T_feet_, feet temperature; T_forehead_, forehead temperature; T_left clav_, left clavicular temperature; T_neck_, neck temperature, T_right clav_, right clavicular temperature.
